# Extracorporeal cardiopulmonary resuscitation for hypothermic refractory cardiac arrests in urban areas with temperate climates

**DOI:** 10.1186/s13049-023-01126-5

**Published:** 2023-10-31

**Authors:** Tal Soumagnac, Jean-Herlé Raphalen, Wulfran Bougouin, Damien Vimpere, Hatem Ammar, Samraa Yahiaoui, Christelle Dagron, Kim An, Akshay Mungur, Pierre Carli, Alice Hutin, Lionel Lamhaut

**Affiliations:** 1 SAMU de Paris-ICU, Necker University Hospital, 149 rue des Sèvres, Paris, 75015 France; 2 Sorbonne University, 21 rue de l’école de médecine, 75006 Paris, France; 3 Jacques Cartier Hospital, 6 avenue du Noyer Lambert, Massy, 91300 France; 4 INSERM U970, Team 4 “Sudden Death Expertise Center”; 56 rue Leblanc, Paris, 75015 France; 5 INSERM U955, Team 3; 1 rue Gustave Eiffel, Créteil, 94000 France; 6 Paris Cité University, 15 rue de l’Ecole de Médecine, Paris, 75006 France

**Keywords:** Hypothermia, ECPR, Cardiac arrest, Prognostic factors

## Abstract

**Background:**

Accidental hypothermia designates an unintentional drop in body temperature below 35 °C. There is a major risk of ventricular fibrillation below 28 °C and cardiac arrest is almost inevitable below 24 °C. In such cases, conventional cardiopulmonary resuscitation is often inefficient. In urban areas with temperate climates, characterized by mild year-round temperatures, the outcome of patients with refractory hypothermic out-of-hospital cardiac arrest (OHCA) treated with extracorporeal cardiopulmonary resuscitation (ECPR) remains uncertain.

**Methods:**

We conducted a retrospective monocentric observational study involving patients admitted to a university hospital in Paris, France. We reviewed patients admitted between January 1, 2011 and April 30, 2022. The primary outcome was survival at 28 days with good neurological outcomes, defined as Cerebral Performance Category 1 or 2. We performed a subgroup analysis distinguishing hypothermic refractory OHCA as either asphyxic or non-asphyxic.

**Results:**

A total of 36 patients were analysed, 15 of whom (42%) survived at 28 days, including 13 (36%) with good neurological outcomes. Within the asphyxic subgroup, only 1 (10%) patient survived at 28 days, with poor neurological outcomes. A low-flow time of less than 60 min was not significantly associated with good neurological outcomes (P = 0.25). Prehospital ECPR demonstrated no statistically significant difference in terms of survival with good neurological outcomes compared with inhospital ECPR (P = 0.55). Among patients treated with inhospital ECPR, the HOPE score predicted a 30% survival rate and the observed survival was 6/19 (32%).

**Conclusion:**

Hypothermic refractory OHCA occurred even in urban areas with temperate climates, and survival with good neurological outcomes at 28 days stood at 36% for all patients treated with ECPR. We found no survivors with good neurological outcomes at 28 days in submersed patients.

**Supplementary Information:**

The online version contains supplementary material available at 10.1186/s13049-023-01126-5.

## Background

Accidental hypothermia designates an unintentional drop in body temperature below 35 °C. Such an event carries the risk of ventricular fibrillation; the risk is major below 28 °C and cardiac arrest is almost inevitable below 24 °C [1] [2]. In such cases, conventional cardiopulmonary resuscitation is often inefficient to obtain return of spontaneous circulation (ROSC).

According to the latest international guidelines, extracorporeal cardiopulmonary resuscitation (ECPR) is considered a potential second line of therapy for hypothermic refractory out-of-hospital cardiac arrest (OHCA) [3]. Refractory cardiac arrest is defined as a failure to obtain ROSC after 20 to 30 min of cardio-pulmonary resuscitation [4]. The Hypothermia Outcome Prediction after ECPR (HOPE) score provides a prediction of the probability of survival in hypothermic OHCA undergoing inhospital ECPR [5] [6]. In Paris’s emergency medical system, ECPR can be implemented in both prehospital or inhospital settings [7]. No study has described patients who have been treated with prehospital ECPR for hypothermic refractory OHCA.

A temperate urban area is characterized by a moderate and mild climate. According to the historical Köppen-Geiger climate classification system [8], these regions generally experience moderate temperature fluctuations between seasons, maintaining relatively comfortable temperatures throughout the year. Temperate climates are not subject to extreme heat or cold, making them less common settings for studies on hypothermia, which is often associated with colder environments. The significance of this study lies in investigating hypothermia in an atypical setting and demonstrating that prognostic outcomes may diverge from those observed in colder regions. Several studies have described the characteristics of patients with hypothermic cardiac arrest in urban areas [9], of patients with hypothermic OHCA treated with ECPR in cold climate settings [10], and of those admitted to a near-alpine-resort trauma center [11]. Because a poor prognosis had already been observed in submersed patients with hypothermic OHCA treated with ECPR [12] [13], we performed a subgroup analysis distinguishing hypothermic refractory OHCA as either asphyxic or non-asphyxic.

## Methods

### Study design

We conducted a retrospective observational monocentric study of patients admitted to Necker University Hospital, a tertiary-care center in Paris, France. We received approval from the ethics committee of the French Society of Anesthesia and Intensive Care Medicine (#IRB 00010254 - 2023–015). Data were collected by a single dedicated individual stationed within the intensive care unit (ICU), responsible for tending to patients throughout the study duration. Research data were obtained from secure medical record sources using computerized and non-computerized databases covering the period of January 1, 2011, to April 30, 2022. The same methodology was consistently employed throughout the study in compliance with French data protection legislation (French Data Protection Authority #MR004_2228701). The inclusion criteria were: an ICD-10 (International Classification Disease, 10th revision) diagnosis of accidental hypothermia (T68); adults aged 18 years or more; prehospital core temperature of 32 °C or less; accidental hypothermia being the primary cause of cardiac arrest, and extracorporeal cardiopulmonary resuscitation with prehospital or inhospital ECPR.

### ECPR protocol

Since 2011, the Service d’Aide Médicale Urgente (SAMU) of Paris has been running an ECPR program [14] [15]. When faced with refractory OHCA cases, the ECMO Team was promptly dispatched to the field. Depending on the estimated duration required to reach the hospital, an on-field decision was taken to either proceed with prehospital or inhospital ECPR. This decision strictly followed a single protocol based on both French [16] and more recent international guidelines [17]. A comprehensive visualization of the ECMO implementation protocol, extracted from French recommendations [16], can be found in the supplemental section of the present study (Figure S1). The protocol remained unchanged throughout the study period.

### Data collection

Water temperatures of the Seine River were collected through the Public Sanitation Service of Paris and were derived from the daily mean obtained from five distinct sanitation stations across the Paris area. They included yearly mean minimum and maximum temperature values from 2011 to 2022 and daily mean temperatures on the specific days when patients were submersed.

Prehospital data collection adhered to the Utstein criteria [18] and included the following variables: age; sex; prehospital external temperature measured using an external thermometer; medical history encompassing alcohol intoxication, drug intoxication, attempted suicide, social precariousness, chronic alcoholism, psychosis, depression and cognitive disorders; assessment of simplified acute physiology score (SAPS II) [19]; HOPE score assessment for patients who underwent inhospital ECPR [5]; initial heart rhythm at the time of OHCA identification; no-flow time defined as the interval spanning from OHCA identification to the initiation of chest compressions, if the precise onset of symptoms could not be determined when the patient was found in OHCA, no-flow time was considered to be greater than 10 min; low-flow time was defined as the interval spanning from the initiation of chest compressions to the initiation of ECPR pumping; epinephrine administration; amiodarone administration and external cardioversion during advanced life support; medical indication for ECPR; and selection of prehospital ECPR implementation or inhospital ECPR implementation.

Inhospital data collection included: central body temperature, which was measured using an oesophageal thermal probe or a urinary catheter equipped with a thermal sensor; pH level upon ICU arrival; serum lactate level upon ICU arrival; serum potassium level upon ICU arrival; administration of norepinephrine and/or administration of dobutamine upon ICU arrival; presence of a disseminated intravascular coagulopathy during hospital stay; use of renal replacement therapy during hospital stay; occurrence of haemorrhagic events during hospital stay meeting the BARC (Bleeding Academy Research Consensus) criterion of overt bleeding associated with a drop in haemoglobin levels of at least 3 to 5 g/dL [20]; incidence of infectious events during hospital stay defined by acute fever associated with an increase in procalcitonin serum levels, necessitating antibiotic treatment with or without microbiological documentation; refractory shock during hospital stay defined as the persistence of haemodynamic shock after ECPR implementation and rewarming; gastrointestinal bleeding; complications attributed to ECPR during hospital stay defined as: scarpa infections requiring surgical intervention, acute limb ischemia, haemorrhagic shock arising from cannulation site bleeding, anoxic encephalopathy, vessel damage requiring surgery; all the above complications occurring before or after 24 h of ICU arrival; length of hospital stay; survival at 28 days; neurological assessment at 28 days according to the Glasgow-Pittsburgh Cerebral Performance Category scale, defined as follows: (1) good cerebral performance, (2) moderate cerebral disability, (3) severe cerebral disability, (4) coma or vegetative state, (5) brain death [21]. The primary outcome was survival at 28 days with good neurological outcome defined as CPC 1 or 2.

We performed a subgroup analysis distinguishing hypothermic refractory OHCA as either asphyxic or non-asphyxic. Hypothermic refractory OHCA resulting from asphyxia was defined as individuals who experienced submersion in water and subsequently developed hypothermia. Non-asphyxic hypothermic refractory OHCA was defined as individuals who developed hypothermia due to exposure to cold air or other non-water-related causes. Ice-cold water was defined as a water temperature below 7 °C. In-house hypothermia was defined as hypothermia occurring indoors, while outside hypothermia was defined as hypothermia occurring outdoors.

### Statistical analysis

Continuous variables were reported as medians with interquartile ranges. Categorical variables were reported as numbers and percentages. Factors associated with favourable neurological outcomes were assessed for categorical variables using the chi-square test or Fischer’s exact test. Continuous variables were divided into categories using relevant thresholds and compared with the chi-square test or Fischer’s exact test. A two-sided p-value of less than 0.05 was considered statistically significant. The statistical analysis was performed using STATA software version v14.0 (Lakeway Drive, TX, USA). Missing data were handled using case-complete analysis.

## Results

**Fig. 1:**
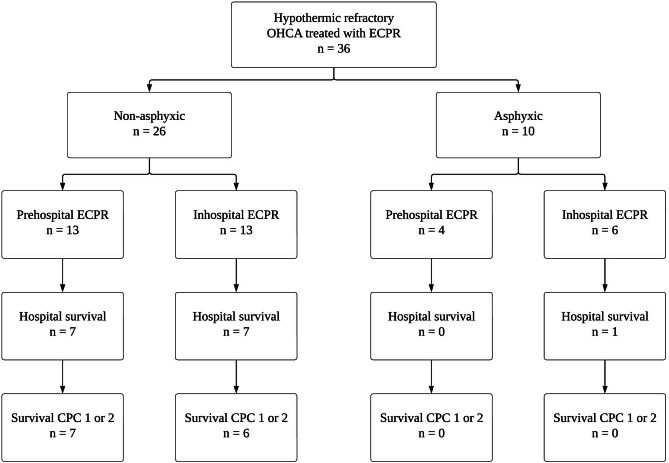
**Patient flowchart.** OHCA, out-of-hospital cardiac arrest; ECPR, extracorporeal cardiopulmonary resuscitation; CPC, cerebral performance category

Between January 1, 2011 and April 30, 2022, a total of 36 patients were included in the study. Among them, 26 were treated with ECPR for hypothermic refractory OHCA due to non-asphyxic causes and 10 were treated for refractory hypothermic OHCA with asphyxic causes following submersion in the Seine River. Out of the 10 submersed patients, only 5 experienced exposure to ice-cold waters below 7 °C, with the lowest recorded temperature on the day of submersion being 3.3 °C (Table S2). The complete patient flow chart is depicted in Fig. 1.

Baseline patient characteristics are displayed in Table 1. The median age was 53 years (IQR 45–66) and 28/36 (78%) patients were male. Upon the arrival of the medical team, the mean external temperature stood at 24.3 °C (IQR 22.0–26.0). Overlapping consumption of various drugs, multiple psychiatric disorders, and social precariousness was observed in our patient population; each patient fulfilled at least one of these criteria. Ventricular fibrillation was the first heart rhythm observed at the scene of cardiac arrest in 23/36 (64%) patients. No-flow time was under 5 min in 14/36 (39%) patients. All 36 patients had a refractory cardiac arrest. Median low-flow time was 83 min (IQR 63–97). Prehospital implementation was carried out in 17/36 (47%) of all patients.

**Table 1:** Patient characteristics before ICU admission

	Total (n = 36)	Non-asphyxic (n = 26)	Asphyxic (n = 10)
Age, *years*	53 (45–66)	55 (55–60)	48 (36–74)
Male	28/36 (78%)	21/26 (81%)	7/10 (70%)
External temperature, *° C*	24.3 (22.0–26.0)	24.4 (22.3–27.1)	24.0 (17.8–25.0)
Context			
* Alcohol intoxication*	15/36 (42%)	11/26 (42%)	4/10 (40%)
* Drug intoxication*	3/36 (8.3%)	2/26 (7.7%)	1/10 (10%)
* Attempted suicide*	6/36 (17%)	1/26 (3.8%)	5/10 (50%)
* Social precariousness*	31/36 (86%)	25/26 (96%)	6/10 (60%)
* In-house hypothermia*	3/36 (8.3%)	3/26 (12%)	0/10 (0%)
* Outside hypothermia*	33/36 (92%)	23/26 (89%)	10/10 (100%)
Chronic psychiatric disorder			
* Alcoholism*	18/36 (50%)	17/26 (65%)	1/10 (10%)
* Psychosis*	5/36 (14%)	4/26 (15%)	1/10 (10%)
* Depression*	9/36 (25%)	5/26 (19%)	4/10 (40%)
* Cognitive disorder*	3/36 (8.3%)	2/26 (7.7%)	1/10 (10%)
SAPS II	86 (77–93)	83 (74–93)	89 (86–93)
Initial rhythm			
* Ventricular fibrillation*	23/36 (64%)	20/26 (77%)	3/10 (30%)
* Asystole*	13/36 (36%)	6/26 (23%)	7/10 (70%)
No-Flow *>* 5 *min*	21/35 (60%)	13/26 (50%)	9/9 (100%)
* Witnessed cardiac arrest*	4/21 (19%)	0/13 (0%)	4/9 (44%)
* Unwitnessed cardiac arrest*	17/21 (81%)	13/13 (100%)	5/9 (56%)
No-Flow time *≤* 5 *min*	14/35 (40%)	13/26 (50%)	1/9 (11%)
Low-Flow time, *min*	83 (63–97)	83 (64–100)	82 (64–96)
Epinephrine administration	36/36 (100%)	26/26 (100%)	10/10 (100%)
Amiodarone administration	10/36 (28%)	10/26 (39%)	0/10 (0%)
External cardioversion, *shocks*	3 (0–7)	5 (2–9)	1 (0–3)
Medical indication for ECPR			
* Refractory cardiac arrest*	36/36 (100%)	26/26 (100%)	10/10 (100%)
ECPR setting			
* Prehospital*	17/36 (47%)	13/26 (50%)	4/10 (40%)
* Inhospital*	19/36 (53%)	13/26 (50%)	6/10 (60%)

Legend: Data are medians (IQR) or numbers (%); SAPS II, Simplified acute physiology score

II; ECPR, Extracorporeal cardiopulmonary resuscitation

Patient characteristics following ICU admission are detailed in Table 2. The overall survival rate at 28 days was 15/36 (42%) of all patients, 14/26 (54%) in the non-asphyxic subgroup and 1/10 (10%) in the asphyxic subgroup. Survival at 28 days with good neurological outcomes, defined as CPC 1 or CPC 2, was observed in 13/36 (36%) of all patients. Within the non-asphyxic subgroup, 13/26 (50%) patients survived with good neurological outcomes, while no survivors were observed in the asphyxic subgroup. Among patients who received prehospital ECPR, 7/17 (41%) achieved survival with good neurological outcomes, whereas in those treated with inhospital ECPR, the rate was 6/19 (32%). Among patients treated with inhospital ECPR, the HOPE score projected a 30% survival rate and observed survival with good neurological outcomes stood at 6/19 (32%).

**Table 2:** Patient characteristics after ICU admission

	Total (n = 36)	Non-asphyxic (n = 26)	Asphyxic (n = 10)
Initial biological parameters			
* pH*	7.10 (6.89–7.32)	7.14 (7.06–7.35)	6.90 (6.75–6.98)
* Lactate level, mmol/L*	10.1 (6.5–18.4)	9.20 (5.5–14.5)	19 (15–24)
* Potassium level, mmol/L*	3.4 (3.1–5.2)	3.7 (3.0-5.1)	3.4 (3.4-6.0)
Inhospital central temperature, °C	25.4 (22.2–29.1)	26.0 (23.0-29.1)	23.2 (16.2–28.4)
Catecholamine administration			
* Maximum norepinephrine at Day 1, mg/h*	9.0 (5.0–20.0)	8.5 (5.8–17.8)	10.0 (5.0–20.0)
* Maximum dobutamine at Day 1, µg/kg/min*	5.0 (5.0–10.0)	5.0 (5.0-8.1)	5.0 (5.0–10.0)
Complications < 24 h			
* RRT*	19/36 (53%)	14/26 (54%)	5/10 (50%)
* Refractory shock*	15/36 (42%)	8/26 (31%)	7/10 (70%)
* Infection*	2/36 (6%)	2/26 (8%)	0 (0%)
* DIC*	31/36 (86%)	21/26 (81%)	10/10 (100%)
* Gastrointestinal haemorrhage*	1/36 (3%)	1/26 (4%)	0/10 (0%)
* Complications related to ECPR (overall)*	3/36 (8%)	3/26 (12%)	0/10 (0%)
* Veino-veinous cannulation*	1/36 (3%)	1/26 (4%)	0/10 (0%)
* Haemorrhagic shock from cannulation site*	2/36 (6%)	2/26 (8%)	0/10 (0%)
* Complications related to inhospital ECPR*	0/19 (0%)	0/13 (0%)	0/6 (0%)
* Complications related to prehospital ECPR*	3/17 (18%)	3/13 (23%)	0/4 (0%)
Complications > 24 h			
* Infection*	12/36 (33%)	10/26 (38%)	2/10 (20%)
* Anoxic encephalopathy*	6/36 (17%)	3/26 (12%)	3/10 (30%)
* Complications related to ECPR (overall)*	8/36 (22%)	7/26 (27%)	1/10 (10%)
* Acute limb ischemia*	0/36 (0%)	0/26 (0%)	0/10 (0%)
* Scarpa infection requiring surgery*	4/36 (11%)	4/26 (15%)	0/10 (0%)
* Vessel damage requiring surgery*	4/36 (11%)	3/26 (12%)	1/10 (10%)
* Complications related to inhospital ECPR*	6/19 (32%)	5/13 (38%)	1/6 (16%)
* Complications related to prehospital ECPR*	2/17 (12%)	2/13 (15%)	0/4 (0%)
Hospital stay, *days*	6 (1–16)	9 (1–17)	1 (1–3)
Survival at 28 days	15/36 (42%)	14/26 (53.9%)	1/10 (10%)
Survival at 28 days with CPC 1/2	13/36 (36%)	13/26 (50%)	0/10 (0%)
* Prehospital ECPR*	7/17 (41.2%)	7/13 (53.8%)	0/4 (0%)
* Inhospital ECPR*	6/19 (31.6%)	6/13 (46.2%)	0/6 (0%)
HOPE Score for inhospital ECPR	30 (4.0–63)	35 (21–71)	2.0 (1.0–16)

Legend: Data are medians (IQR) or numbers (%), CPC, Cerebral performance category; ECPR, Extracorporeal cardiopulmonary resuscitation; RRT, Renal replacement therapy; DIC, disseminated intravascular coagulation

Table 3 presents an analysis of factors associated with good neurological outcomes at 28 days. The absence of asphyxia was significantly associated with good neurological outcomes (P < 0.01) in contrast to asphyxia. A no-flow time of less than 5 min was also significantly associated with good neurological outcomes (P < 0.01). Conversely, a low-flow time of less than 60 min was not associated with good neurological outcomes (P = 0.25). Similarly, we observed no statistically significant difference in survival rates between patients treated with prehospital ECPR 7/17 (41%) compared with patients treated with inhospital ECPR 6/19 (32%) (P = 0.55).

**Table 3:** Factors associated with good neurological outcomes at 28 days

	Favorable CPC 1–2	Non favorable CPC 3–5	P-value for interaction
Type of exposure			
*Non-asphyxic*	13/26 (50%)	13/26 (50%)	< 0.01
*Asphyxic*	0/10 (0%)	10/10 (100%)	
ECPR setting			
*Prehospital*	7/17 (41%)	10/17 (59%)	0.55
*Inhospital*	6/19 (32%)	13/19 (68%)	
No-flow duration			
*≤ 5 min*	9/13 (70%)	5/22 (23%)	< 0.01
*> 5 min*	4/13 (30%)	17/22 (77%)	
Low-flow duration			
*≤ 60 min*	9/13 (70%)	18/21 (86%)	0.25
*> 60 min*	4/13 (30%)	3/21 (14%)	

Legend: Data are numbers (%), CPC, Cerebral performance category; ECPR, Extracorporeal cardiopulmonary resuscitation

## Discussion

Within our study cohort, 13/36 (36%) patients achieved survival with good neurological outcomes at 28 days, including 13/26 (50%) patients in the non-asphyxic subgroup. However, we did not observe any instances of survivors with good neurological outcomes at 28 days among submersed patients. Prehospital ECPR showed no statistically significant difference in survival with good neurological outcomes when compared with inhospital ECPR (P = 0.55). Prolonged low-flow was not found to be associated with poorer neurological outcomes (P = 0.25). In patients treated with inhospital ECPR, survival predicted by the HOPE score was 30% and observed survival was 6/19 (32%).

Similar survival rates were reported in various studies across different climatic regions. These rates included a 55% survival rate in a continental climate area [9], a 26.5% survival rate in an oceanic climate area [12], a 20% survival rate in an alpine-sport-related accident setting [11], and a 100% survival rate in a high desert climate [22]. It’s important to note that our study population differed from those in these studies. Indeed, a significant portion of our patients faced social precariousness, and/or psychiatric disorders including alcoholism in 18/36 (50%) cases, drug use in 3/36 (8%) cases, psychosis in 5/36 (14%) cases and depression in 9/36 (25%) cases. A study conducted in France between 2000 and 2010, spanning 18 urban areas, attributed 3.9% of all deaths to cold exposure [23]. Notably, deaths related to hypothermia were nearly ninety times more frequent among socially precarious individuals compared with the general population [24]. Additionally, another study conducted in Paris concerning addictions and psychiatric conditions among socially distressed individuals reported that 31.5% suffered from psychiatric disorders [25]. In alignment with these findings, a substantial majority of our patients, 31 out of 36 (86%), faced social precariousness, and 29 out of 36 (81%) presented with at least one psychiatric disorder. This specific profile of accidental hypothermia patients in urban areas with temperate climates represents a significant confounding factor. Therefore, caution must be exercised when comparing hypothermia patient cohorts, and the influence of this parameter should not be overlooked.

We established a significant correlation between the occurrence of asphyxia resulting from submersion in patients experiencing hypothermic refractory OHCA and an increased mortality rate (P < 0.01). Notably, within the asphyxic subgroup, there were no survivors who achieved good neurological outcomes. These findings concur with previous research [12] [26] [27], particularly a study conducted in Paris from 2002 to 2012 that concentrated on inhospital ECPR for drowned patients. In that particular study, out of 20 drowned patients treated with ECPR, only 1 patient was discharged from the hospital with good neurological outcomes, while another patient had poor neurological outcomes [13]. In our study, which encompassed patients treated with both inhospital and prehospital ECPR, only a single patient survived to hospital discharge with poor neurological outcomes. Several hypotheses have been formulated to elucidate these findings. Firstly, certain publications have posited that the neuroprotective effect of water is exclusively conferred by ice-cold water (< 7 °C) [28]. In our investigation, we meticulously recorded the temperature of the Seine River on the day of submersion to explore this threshold. Among the 10 submersed patients, only 5 were exposed to temperatures below 7 °C. The temperatures recorded on the day of submersion in the Seine River ranged from as low as 3.3 °C to as high as 23.5 °C. When considering the comprehensive temperature data of the Seine River (as presented in Table S1 and Table S2) during submersion incidents, the often elevated water temperatures exceeding 7 °C, along with the resulting loss of their neuroprotective attributes, offer a compelling explanation for the heightened mortality observed in our study within the Paris area. Secondly, other noteworthy distinctions highlighted in Tables 1 and 2 can account for the mortality difference between our subgroups. For instance, the asphyxic subgroup exhibited an increased prevalence of asystole 7/10 (70%) compared with the non-asphyxic subgroup 6/26 (23%). Also, the duration of no-flow was more often higher than 5 min in the asphyxic subgroup 9/10 (90%) compared with the asphyxic subgroup 10/26 (39%). These outcomes warrant a reconsideration of the criteria for ECPR, whether conducted in-hospital or prehospital, for hypothermic patients with refractory OHCA following submersion within urban areas with temperate climates.

Regarding prehospital ECPR, a previous study has suggested that the implementation of ECPR in a prehospital setting might offer advantages over inhospital ECPR for OHCA [7]. However, this matter remains ambiguous, as only a few medical teams practice prehospital ECPR, and no large, randomized clinical trial has yet addressed this question. In our present study, we observed no statistically significant difference in survival rates with good neurological outcomes between prehospital ECPR 7/17 (41%) and inhospital ECPR 6/19 (32%) (P = 0.55). However, the size of our sample indicates that the analysis of this statistical result must be approached with great caution. In any case, one of the suggested explanations that could account for this outcome is the less discriminative nature of low-flow in patients with hypothermic refractory OHCA compared with those with non-hypothermic refractory OHCA [29]. Indeed, the median low-flow time in our study reached 83 min (IQR 63–97), surpassing the typical 60-minute threshold linked to survival in ECPR [15]. Surprisingly, a low-flow duration of 60 min or less did not exhibit a statistically significant correlation with favorable neurological outcomes at 28-days (P = 0.25). This outcome prompts doubts about the applicability of the 60-minute implementation guideline for the specific subset of patients encountering hypothermic refractory OHCA. In contrast, a no-flow duration of less than 5 min displayed a statistically significant association with positive neurological outcomes (P < 0.01). As a result, while the duration of no-flow remains of utmost importance, the significance of low-flow might be diminished in cases of hypothermic refractory OHCA [30], thereby reducing the advantage of prehospital ECPR compared with other indications.

The HOPE score, introduced in 2018, estimates the survival probability of patients admitted to the ICU after experiencing hypothermic OHCA and undergoing inhospital ECPR [5]. Due to the frequent absence of serum potassium levels in the prehospital setting, our score calculations were limited to patients receiving inhospital ECPR. Within this particular group, our analysis revealed a projected survival rate of 30% as predicted by the HOPE score. Similarly, the actual observed survival rate with good neurological outcomes at 28 days was recorded at 6/19 (32%). Nevertheless, the utility of the score could prove advantageous in urban areas with temperate climates for effectively prioritizing hypothermic patients in both prehospital and inhospital scenarios.

### Limitations

We wish to acknowledge several limitations in our study. Firstly, the size of our participant group remained modest. Despite the rarity of the phenomenon we were exploring, having a larger number of patients in our study would have improved the statistical strength of our results. This would have been especially valuable for conducting more precise comparisons between prehospital and inhospital ECPR survival rates, as well as for facilitating more effective examinations of various subgroups.

Secondly, due to the retrospective nature of our research and our reliance on medical records, we encountered missing data, notably in relation to electroencephalography, chest computed tomography, and brain imaging. However, it is essential to consider that the patients in our study, as reflected in their prognosis, were highly critical cases following hypothermic refractory OHCA treated with ECPR and were seldom feasible for transport to the Radiology department in such conditions.

An additional constraint was the method used for measuring prehospital external temperatures, which involved an external thermometer. Unfortunately, this method’s reliability was not ideal for accurately representing the actual core temperature. Furthermore, in three cases, we faced challenges where the external thermometer failed to detect extremely low temperatures, making the prehospital external temperature data unavailable. In these situations, we relied on the clinical assessment of hypothermia, which we supplemented with central temperature measurements upon the patients’ admission to the ICU. It is worth noting that this central temperature data did not have any missing values and consistently supported the presence of significant hypothermia in all patients.

## Conclusions

Hypothermic refractory OHCA occurred even in urban areas with temperate climates, and survival with good neurological outcomes at 28 days stood at 36% for all patients treated with ECPR. We found no survivors with good neurological outcomes at 28 days in submersed patients.

### Electronic supplementary material

Below is the link to the electronic supplementary material.

Supplementary Material 1

Supplementary Material 2

Supplementary Material 3

## Data Availability

The datasets used and/or analysed during the current study are available from the corresponding author on reasonable request.

## Abbreviations

ECPR: Extracorporeal Cardiopulmonary Resuscitation
ROSC: return of spontaneous circulation
HOPE: Hypothermia Outcome Prediction after ECPR
ICU: Intensive Care Unit
SAMU: Service d’aide médicale Urgente
SAPS II: Simplified Acute Physiology Score II
RRT: Renal Replacement Therapy
DIC: Disseminated Intravascular Coagulopathy
BARC: Bleeding Academy Research Consensus
