# Supplementary material for: Extracorporeal cardiopulmonary resuscitation for hypothermic refractory cardiac arrests in urban areas with temperate climates
Source: Scand J Trauma Resusc Emerg Med. 2023 Oct 31;31:68. doi: 10.1186/s13049-023-01126-5 (PMC10619216; doi:10.1186/s13049-023-01126-5)

**Table S1.** Mean daily temperatures of the Seine River (°C)

|  | Minimum | Maximum |
| --- | --- | --- |
| 2011 | 2.40 | 22.90 |
| 2012 | 1.69 | 23.96 |
| 2013 | 4.26 | 25.88 |
| 2014 | 6.45 | 23.99 |
| 2015 | 4.30 | 25.53 |
| 2016 | 5.20 | 23.36 |
| 2017 | 3.18 | 26.19 |
| 2018 | 3.03 | 26.46 |
| 2019 | 5.20 | 26.98 |
| 2020 | 6.62 | 26.34 |
| 2021 | 2.61 | 24.28 |
| 2022 | 4.69 | 25.93 |
|  |  |  |
| Legend: Values are means (°C) from five wastewater treatment plants located on the Seine River in the Paris area: Suresnes, Bougival, Andrésy, Méricourt and Alfortville. |  |  |
|  |  |  |
|  |  |  |
|  |  |  |
|  |  |  |
|  |  |  |
|  |  |  |

**Table S2.** Mean temperature of the Seine River on the day of submersion (°C)

| Temperature | | Minimum  (2000-2022) | Maximum  (2000-2022) |
| --- | --- | --- | --- |
|  | |  |  |
| 08/06/2013 | 17.75 | 23.02 | 15.88 |
| 17/07/2013 | 23.46 | 25.35 | 17.63 |
| 17/12/2013 | 5.41 | 9.17 | 3.08 |
| 25/01/2017 | 3.34 | 8.80 | 3.34 |
| 15/12/2016 | 6.32 | 9.35 | 3.95 |
| 19/11/2017 | 9.87 | 13.61 | 7.14 |
| 29/01/2018 | 8.82 | 9.09 | 3.33 |
| 14/02/2018 | 5.15 | 9.85 | 1.95 |
| 17/01/2019 | 7.43 | 9.04 | 3.26 |
| 28/01/2020 | 6.72 | 8.92 | 3.18 |

Legend: Values are means (°C) from five wastewater treatment plants located on the Seine River in the Paris area: Suresnes, Bougival, Andrésy, Méricourt and Alfortville.


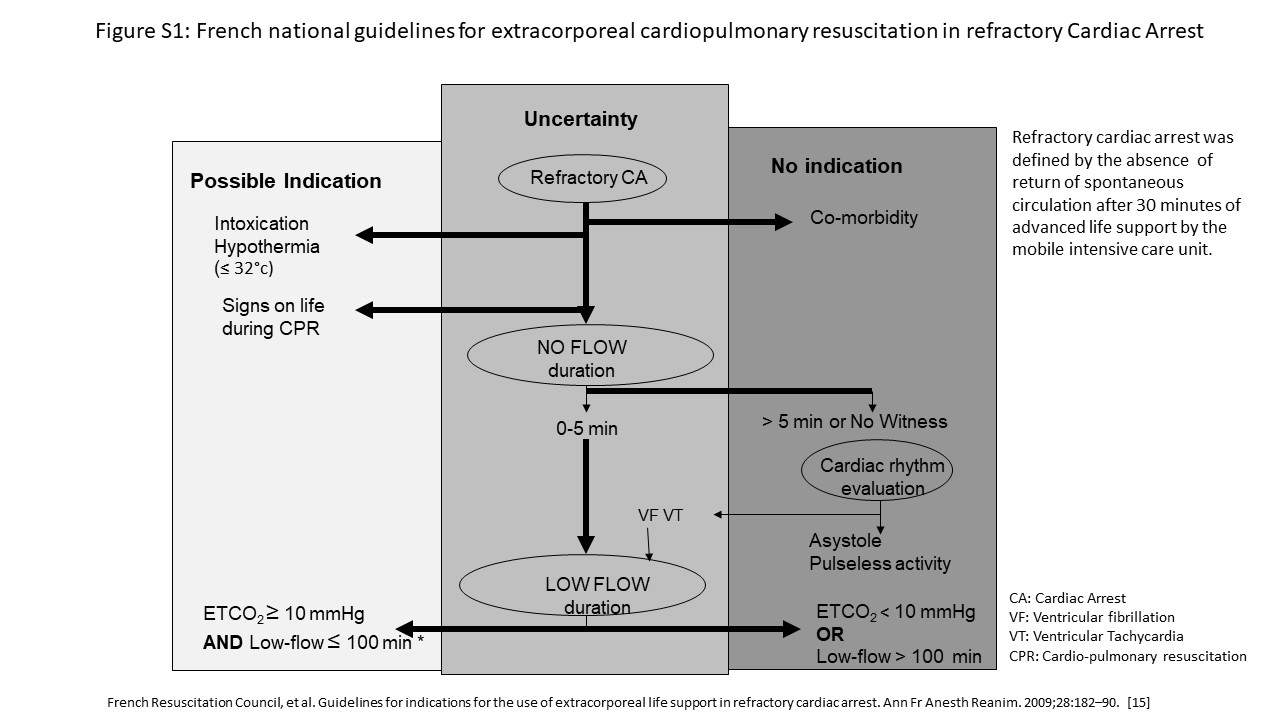

Supplement: Supplementary file 1 — Supplementary Material 1 [file 13049_2023_1126_MOESM1_ESM.docx]
