# Supplementary material for: Extracorporeal cardiopulmonary resuscitation for hypothermic refractory cardiac arrests in urban areas with temperate climates
Source: Scand J Trauma Resusc Emerg Med. 2023 Oct 31;31:68. doi: 10.1186/s13049-023-01126-5 (PMC10619216; doi:10.1186/s13049-023-01126-5)
Supplement: Supplementary file 2 — Supplementary Material 2 [file 13049_2023_1126_MOESM2_ESM.docx]

Lionel Lamhaut MD PhD

Hospital Necker

SAMU de Paris

149 Rue de Sevres

Paris 75015

France

[Lionel.lamhaut@aphp.fr](mailto:Lionel.lamhaut@aphp.fr)

Editor

Scandinavian Journal of Trauma, Resuscitation and Emergency Medicine

**Re: Hypothermic Out-of-Hospital Cardiac Arrests in Temperate Urban Areas Treated with Prehospital or In-hospital ECPR**

March 24, 2023

Dear Editor-in-Chief,

We are pleased to submit our original paper entitled "Hypothermic Out-of-Hospital Cardiac Arrests in Temperate Urban Areas Treated with Prehospital or In-hospital ECPR" for consideration for publication in Scandinavian Journal of Trauma, Resuscitation and Emergency Medicine.

To our knowledge, this is the first article to investigate refractory hypothermic cardiac arrest in a temperate urban area such as Paris. Additionally, it is the first series that includes patients resuscitated with prehospital ECPR. With ECPR becoming more widespread, teams will need to know if it is relevant for refractory hypothermic cardiac arrest in temperate urban areas, not just in cold settings, and whether ECPR shows good outcomes with this indication.

On behalf of the authors, I declare that all have significantly contributed to the conception and design of the paper, reviewed drafts, agreed on the final version of the paper, and agreed to be accountable for all aspects of the work of the paper submitted for consideration.

I further declare that this work has not been published in any other journal, nor is the manuscript under consideration elsewhere.

We would like to thank you for your consideration and look forward to hearing about the article's suitability for publication in your journal.

Sincerely,

Pr Lionel Lamhaut, MD, PhD
