# Supplementary material for: Extracorporeal cardiopulmonary resuscitation for hypothermic refractory cardiac arrests in urban areas with temperate climates
Source: Scand J Trauma Resusc Emerg Med. 2023 Oct 31;31:68. doi: 10.1186/s13049-023-01126-5 (PMC10619216; doi:10.1186/s13049-023-01126-5)
Supplement: Supplementary file 3 — Supplementary Material 3 [file 13049_2023_1126_MOESM3_ESM.docx]

**Dear Reviewers, all the authors involved in this article would like to thank you for the quality and the relevance of your comments. We hope to have been able to provide answers to all your questions and remain at your disposal to address any further comments and suggestions.**

**Reviewer #1: Thank you for the opportunity to review this interesting and well-written paper on Hypothermic out-of-hospital cardiac arrests. I enjoyed reading it. This topic is interesting for professionals of medical emergency services who work in the EMS/ER and ICU. I have no major issues with this paper. I have read it thoroughly several times.**

We would like to thank the reviewer for this comment**.**

**Of minor issues, I would like to bring out the following:**

**Regarding the cold-water exposure group; the pathophysiology and prognosis clearly differ between the group of asphyxia and subsequent secondary hypothermia and the primary hypothermia in cold water exposure. According to the methods section, these (line 93) secondary CA would be excluded; however, they are then mentioned and discussed in line 224. Are chest CT images available at ER admission to better differentiate this group? Include this aspect in the limitations.**

Presently, the existing French guidelines concerning the eligibility for ECPR subsequent to hypothermic cardiac arrest do not differentiate between patients who become hypothermic due to cold air exposure (non-asphyxic group in the revised manuscript) and those who experience hypothermia because of cold-water submersion (asphyxic group in the revised manuscript). Drawing from our clinical insights gained within the urban area of Paris, we hypothesized a potential prognostic discrepancy between these distinct cohorts. Consequently, a primary focal point of our study was to illustrate the significant contrast in terms of survival outcomes that manifests between these two different populations.

In the initial manuscript, specifically at line 224, we endeavored to expound upon the reasons for the distinctive prognosis of our submersed patients in comparison to prior studies addressing this inquiry; however, this passage lacked scientific rigor. Other distinctions observed in Tables 1 and 2, such as the increased prevalence of asystole in the cold water group (asphyxic group in the revised manuscript) and the extended duration of no-flow, offer scientifically more robust evidence to elucidate the disparity in mortality between the two groups. Furthermore, considering the reviewer's #2 comments and the amassed data concerning the Seine River's temperature at the time of submersion, the warmth of the water, often above 7°C, along with the loss of its neuroprotective attributes, seems to provide another more compelling argument.

We have extensively revised the manuscript to address the comments of both reviewers on this matter.

Regarding the CT scan data, we conducted a re-evaluation of our database, and information regarding thoracic CT scans was not accessible upon admission to the intensive care unit. However, upon arrival in the intensive care unit, all patients underwent a chest X-ray and a FAST ultrasound in addition to the analysis of their medical history, which did not reveal any associated trauma. These tests were performed to exclude significant thoracic injuries separate from those that might be linked to prolonged cardiac massage. A statement has been added to the limitations section of the revised manuscript, acknowledging the absence of CT scan data for all patients. Furthermore, it is important to bear in mind that submersed patients, as indicated by their prognosis, were highly critical cases following refractory cardiac arrest treated with ECPR, and were rarely transportable in such conditions to the Radiology department.

**Line 151 & Tab 2: in 46% no-flow time was less than 5min; what was the proportion of observed and unobserved CA in the >5min no-flow group?**

We have corrected this inaccuracy in both Table 1 and the manuscript. We have incorporated the overall count of witnessed cardiac arrests, along with the specific counts of witnessed cardiac arrests among patients with a no-flow time of less than 5 minutes and greater than 5 minutes.

**Tab 1&2 and perhaps 3 could be summarized.**

In accordance with this comment, we have merged Tables 1 and 2. To enhance clarity, we have maintained Table 3 as a separate entity.

**Fig. 2 Ambiguity of x axis labelling. Perhospital/inhospital (missing ECPR strategy? Or what does it refer to?).**

After making significant changes to the manuscript, we realized that this simplified figure does not adequately represent the nuances and distinctions among the groups. Consequently, we have opted to exclude it from the manuscript.

**Reviewer #2: Thank you for the honor and pleasure to review the manuscript Hypothermic out-of-hospital cardiac arrest in temperature urban areas treated with prehospital and in-hospital ECPR. The study summarizes 11 years of ECPR experiences with 37 patients. Comparisons were made between persons who had become cold in air and water and between ECPR started out-of-hospital or in-hospital. This is a relevant study that will be read with great interest by supporters and criticasters of ECPR in pre-hospital resuscitation setting. As such, the manuscript should be as unambiguous and clear as possible. At this moment, there are still many loose ends in the manuscript to provide robust arguments in the on-going discussion.**

We would like to thank the reviewer for this comment**.**

**l. 87-94 - it is advised that the authors add information that allows a better understanding of the quality of data retrieval, collection, and processing. For example: was this done by one or two persons; please describe which procedures were taken that the quality of all data at the basis of this study can be considered adequate and reliable.**

As added in the methodology section of the manuscript, data collection was conducted by a dedicated individual working within the intensive care unit responsible for attending to patients during the study period. Research data were obtained from secure medical record sources and were securely stored on a computer. The same methodology was consistently employed throughout the study and received ethical approval from an ethical committee, the number of which has been included in the methods section.

**l. 97-101 - please clarify the procedure regarding the decision for ECPR or not and were the decisions always taken according to existing guidelines and protocols.**

The ECPR program of the Service d’Aide Médical d’Urgence (SAMU) of Paris was established in 2011 with the creation of a dedicated mobile intensive care unit (ICU), known as the ECMO Team. Between 2011 and 2022, when a patient experienced refractory OHCA following accidental hypothermia, the ECMO Team was dispatched. Depending on the time required to reach the hospital, a decision was made to perform prehospital or inhospital therapeutic ECPR following a strict protocol, which was based on French recommendations.

We have provided further details regarding this matter in the methods section of our manuscript. Additionally, we have included a figure summarizing the ECMO implementation protocol extracted from French recommendations in the supplemental section of the manuscript (Figure S1).

**Clarify the difference between procedures, guidelines, protocols, management, and strategies. If these are different words for the same issue, please use one word consistently. If not, please clarify the differences and use also the different words in a consistent manner. This also refers to the results in lines 152-153 and 213-223.**

We have rectified this inaccuracy by using the same term throughout the manuscript. The chosen term was "protocol," as it refers to the protocols mentioned in the previous comment.

**Please mention whether the guidelines and protocols have changed, or not, over the eleven years of the study. It may be considered to add the (several) procedures / protocols in an addendum.**

The protocol remained unchanged throughout the study period. We mentioned it in the manuscript. As mentioned in previous comments, we have also included relevant references and a figure derived from the French recommendations that provides an overview of this protocol (Figure S1).

**l. 104-110 - please clarify if and how central body temperature was measured and refer to the reliability of temperature measurements in the discussion as well as limitation section of the manuscript.**

The initial temperature measurement recorded in Table 1 represents the prehospital external temperature, captured using an external thermometer. We have refined the label for this entry in Table 1 to enhance clarity.

Furthermore, within the same table, we have introduced an additional entry detailing the central temperature measurement taken upon admission to the intensive care unit. This subsequent temperature reading was measured using either an esophageal thermal probe or a urinary catheter equipped with a thermal sensor.

For three patients, the pre-hospital external temperature was unavailable due to exceedingly low values that remained undetected by the external thermometer. In these cases, the clinical diagnosis of hypothermia was supplemented by central temperature measurement upon arrival in the intensive care unit. Notably, this central temperature data exhibited no missing values and consistently affirmed the presence of profound hypothermia. We have acknowledged this limitation regarding the reliability of initial external temperature measurement in the manuscript's limitations section.

**Provide more details how no-flow time and low-flow time have been defined, including the definition of the start and finish of these time (also referring to the text in l. 168-170 and table 4).**

No-flow time was defined as the interval spanning from cardiac arrest to the initiation of chest compressions. If the patient was found in cardiac arrest without the possibility of precisely determining the onset of symptoms, the no-flow period was considered to be greater than 10 minutes. Low-flow time was characterized as the interval spanning from the initiation of chest compressions to the onset of ECPR pumping. These definitions have been incorporated into the methods section of the manuscript.

**More details on the dosages of epinephrine, norepinephrine, amiodarone and dobutamine (table 3) are needed to understand the treatment characteristics.**

We have provided the details regarding the dosages of the aforementioned medications in Table 2 of the manuscript.

**It is not clear if the complications upon arrival at the ICU relate to complications existing at the moment of ICU admission or to complications that occurred after the hypothermic patient had been in the ICU for some time. This distinction may be relevant to understand the direct, delayed or ECPR-related complications of deep hypothermia.**

In order to address this comment, we differentiated in Table 2 between complications occurring within the first 24 hours after admission to the intensive care unit and those occurring after 24 hours. For each section (less than 24 hours and more than 24 hours), we reported the complications related to ECPR. We have also defined in the methods section of the manuscript what was considered as a complication attributed to ECPR.

**l. 124-125 - a major element of the study seems to be aimed at the understanding of the distinction between hypothermia as a result of cold air and cold water (see also later). For the correct understanding of the circumstances, it may be relevant to describe in more details what has been the definition of cold air and cold water.**

To better align with the terminology used in the current scientific literature, we modified the group names to “asphyxic” and “non-asphyxic”

The asphyxic group included individuals who experienced submersion in water and subsequently developed hypothermia. The non-asphyxic group included patients who developed hypothermia due to exposure to cold air or other non-water-related causes.

We added these definitions in the methods section of the manuscript.

**Several publications mention that only ice-cold water (below 7oC) is protective. Have all persons who drowned in cold water indeed entered ice-cold water?**

To address this comment, we established contact with the Public Sanitation Service of Paris, which provided us with daily average water temperature values for the Seine River. These values were derived from daily averages obtained from five distinct sanitation stations across the Paris region: Suresnes, Bougival, Andrésy, Méricourt, and Alfortville.

As a result, to provide the reader with a general idea of the temperatures of the Seine River, we have included a supplementary table in the manuscript, presenting the yearly average minimum and maximum temperature values throughout the study period. Additionally, another table showcases the daily average temperatures on the specific days when patients were submersed. By utilizing this dataset, we can confirm that 5 patients were exposed to water temperatures below 7°C during the study period.

We have addressed this issue comprehensively in the methods section of the manuscript, further elaborated on it in the results section of the manuscript, and engaged in a discussion of it in the discussion section of the manuscript.

**It is also suggested to differentiate between in-house and outside situations as there generally will be differences in speed and duration of hypothermia. The authors need to consider if it is a limitation that that two groups with different "temperature profiles" are put together.**

We have rectified this ambiguity by adding two lines to Table 1 in order to distinguish between in-house hypothermia and hypothermia occurring outside. Among the patients included in our study, 3 patients experienced in-house hypothermia, with 1 patient found with windows wide open. The limited number of in-house cases leads us to believe that grouping them together did not represent a significant confounding factor for the study statistical analysis.

**l. 171-174 - it should be considered that the several absences of a statistically significant difference are due to the small group of included patients. Maybe a power analysis is not correct or possible, but this would give an indication how many persons should have been included to find a statistically significant difference. It is advised to use statistics sparingly in this manuscript.**

To address this comment, we conducted a theoretical calculation of the required sample size for comparing two binomial variables at 44% versus 30%, using a two-tailed test with a 5% Type I error rate and 90% power. The necessary number of subjects to detect this difference as statistically significant is 496 patients, with 248 patients in each group. However, we chose not to include this information in the study because its primary objective is not to compare the performance of prehospital ECMO to in-hospital ECMO. Additionally, we have emphasized in the limitations section that the sample size is a significant constraint when comparing the inhospital and prehospital subgroups.

**l.177- etc - it is suggested starting the discussion with all the major findings of the study and refer to relevant literature later in the manuscript. The number of recent (after f.e. 2018) references to other significant studies on this issue is limited, leading to a rather basic discussion. Both the study objective and the methodology have major points that may deserve more in-depth attention and existing evidence in the discussion. Some controversies on the topic have been published by the same author.**

We have reorganized the discussion to commence with all the major findings of our study. Furthermore, we have conducted a search for more up-to-date references relevant to the topics addressed in the discussion, aiming to augment its depth and comprehensiveness.

**l. 182-190 - it is advised trying to summarize the relevancy of the findings from these studies in one sentence. It is not needed to mention names of authors in the main text. This is evident from the references.**

We attempted to summarize the relevance of the findings from these studies in a single sentence. Additionally, as suggested, we have removed the authors' names from the text.

**l. 230-232 - the inclusion of patients with severe, and most likely lethal, injuries in this series of cold water drowning can be considered a major confounder. Their inclusion also does not match with the stated inclusion criteria (l. 93: "accidental hypothermia being the primary cause of cardiac arrest). It should be a serious consideration to exclude these patients. The inclusion of this category will most likely have blurred data regarding f.e. the needs for inotropics, presence of hemorrhagic events (100% in this study but not previously reported in any drowning study) and DIC. Information on the trauma score seems relevant to be mentioned.**

Indeed, traumatic cardiac arrests did serve as an exclusion criterion in our study. Upon reevaluation of all medical records, which encompassed patient histories, clinical examinations, and available imaging, and through the meticulous review by two different reviewers, we found one patient whose history was consistent with a fall from a height of 4 meters and a possible traumatic context. This patient was excluded from the study. All the statistics were then recalculated accordingly. For the 36 remaining patients, there was no indication of a traumatic confounding factor, and no evidence of falls was found in the prehospital medical personnel's records.

The specific sentence (l. 230-232) initially formulated as a hypothesis regarding the survival disparity between the submersed patient group and those exposed to cold air was more of a conjecture arising from the absence of an evident explanation for this difference, rather than being grounded in the objective data of our study. It clearly lacked scientific rigor and we removed it from the manuscript.

Additionally, other distinctions observed in Tables 1 and 2, such as the increased prevalence of asystole in the cold water group and the extended duration of no-flow, offer scientifically more robust evidence to elucidate the disparity in mortality between the two groups. Finally, considering the amassed data concerning the Seine River's temperature at the time of submersion, the warmth of the water above 7°C, along with the loss of its neuroprotective attributes, seems to provide another more compelling argument. We have extensively discussed these findings in the discussion section.

**When comparing the cold air and cold water characteristics in tables 1 and 2, there are also some other differences that suggest that both groups are very different. These differences may also be a not-mentioned reason why the drowned hypothermic patients had such a bad outcome. In other words, in this study the concomitant factors may have more influenced the outcome than the difference between cold water and cold air. It may also be considered that there are several reason why it is technical more difficult to start ECPR in drowned persons.**

We agree with this comment and have provided several additional explanations regarding the prognostic differences between our two groups (see previous comment). Indeed, one of the primary objectives of our study was to emphasize the concurrent factors associated with drowning and to illustrate that these distinctions, within the temperate urban area of Paris, result in unfavorable prognoses for submersed patients, potentially precluding them from ECPR indication.

We acknowledge that this objective lacked clarity in our manuscript and have undertaken significant revisions to emphasize its significance more effectively.

**It is in this part of the discussion that relevant, and more recent, references regarding ECPR in drowning (both from studies and reviews) are useful to place into perspective the observation from this study of the bad prognosis when ECPR used in drowning. On the other hand, few studies have compared air and water hypothermia. Previous studies that compared drowned and non-drowned hypothermic patients (but not treated by ECPR) found a better outcome in drowning hypothermic persons.**

We have added to the manuscript more relevant and recent references regarding ECPR in drowning and discussed the specifications of our populations compared to previous studies.

**l.249-250 - with this small number of patients, it must be not too difficult to collect data on the outdoor and water temperatures on the day of admission of each patient. This would provide more insight than referring to a crude measure as season.**

To address this comment, we established contact with the Public Sanitation Service of Paris, which provided us with daily average water temperature values for the Seine River. These values were derived from daily averages obtained from five distinct sanitation stations across the Paris region: Suresnes, Bougival, Andrésy, Méricourt, and Alfortville.

As a result, to provide the reader with a general idea of the temperatures of the Seine River, we have included a supplementary table (Table S1) in the manuscript, presenting the yearly average minimum and maximum temperature values for the Seine River throughout the study period. Additionally, another table (Table S2) showcases the daily average temperatures on the specific days when patients were submersed.

**l.254-260 - it is not clear how relevant these sentences are for the study. And if so, if the essence of the sentences should be part of the limitations.**

Considering this feedback, we concur that these sentences lack significant relevance to the manuscript. Consequently, they have been removed from the revised version of the manuscript.

**l.261-268 - this may have a separate heading "limitations". This review mentions several other limitations that need to be addressed.**

We have added a separate heading “limitations” to the manuscript and addressed the limitations mentioned in the review.

**The manuscript will certainly benefit from editing and restructuring of content by a native English speaker with an academic background in health research. It would make the manuscript more granular when the comparisons between persons who had become cold in air and water and between ECPR started out-of-hospital or in-hospital were better structured.**

We enlisted the services of a native English speaker with an academic background in health research to edit and restructure the manuscript.

**Some details**

**The title of the manuscript is confusing. Please provide a clear title that allows immediate understanding about the topic of the study. Also, abbreviations should be avoided in a manuscript title.**

We have updated the manuscript title to:

"Extracorporeal Cardiopulmonary Resuscitation for Hypothermic Cardiac Arrests in Urban Areas With Temperate Climates."

**It should be considered that the term "temperature urban areas" is rather uncommon. It needs more explanation why this setting has a relevant added value for the study, including explaining the impact of an urban area on colder temperatures. In general, urban environments are recognized as environments with higher temperature.**

A temperate urban area is characterized by a moderate and mild climate. These regions generally experience moderate temperature fluctuations between seasons, maintaining relatively comfortable temperatures throughout the year. Temperate climates are not subject to extreme heat or cold, making them less common settings for studies on hypothermia, which is often associated with colder environments. The significance of this study lies in investigating hypothermia in an atypical setting and demonstrating that prognostic outcomes may diverge from those observed in colder regions.

We have added these explanations in the introduction section of the manuscript.

**l.57 - please clarify what is meant by "unpredicted" and why this word is relevant.**

We agree with this comment that the added value of this term is not evident, so we have removed it from both the abstract and the manuscript and replaced it with “unintentional”.

**l. 60-62 - this sentence may better fit after reference 4 in line 66.**

We have moved this sentence within the manuscript, after reference 4.

**l. 76 - what is meant by "this indication"**

It is meant that no study specifically describes patients treated with prehospital ECPR for refractory hypothermic cardiac arrest. We have clarified this point in the manuscript.

**l.79 - there is international consensus that the term "near-drowning" is not used. Current terminology is fatal and non-fatal drowning. This includes both submersion and immersion. It is not clear from the current descriptions what the categories were of the included drowned persons.**

This is an inaccuracy on our part. We have replaced the term "near-drowning" with "submersed" in the manuscript.

**l. 87 - please add the information regarding ethical approval (registration number) as mentioned in l.306**

We added the information regarding ethical approval obtained from the ethics committee of the French Society of Anesthesia and Intensive Care Medicine (#IRB 00010254 ‐ 2023 - 015).

**l.97 - this seems more a protocol than a strategy (see also remark above)**

We changed the term strategy by the term protocol in the text.

**l.128 - the "we-form" should be avoided here.**

We have removed the "we-form” from this line of the text**.**

**l.152-153 - not clear what is meant here and if, twice mentioned, 17/37 relate to the same data information.**

We have the same proportion of patients who received prehospital ECMO and the same proportion of patients with a no-flow time less than 5 minutes. Therefore, this proportion appears twice in the text.

**l.273 - the italic Conclusion can be removed.**

The italic Conclusion was removed from the text.

**References**

**As mentioned above, there are recent studies that provide information relevant in the context of this manuscript.**

**l. 372, 373 - Ref [1] and Ref [2]. There are several more recent excellent reviews and updates on accidental hypothermia than these references from 1994 and 2012**

As suggested, we have updated these references to more recent reviews on accidental hypothermia**.**

**l. 375 - Ref [3] - please also provide the English translation**

We have provided the English translation for this reference.

**l. 419 - Ref [19] - It may be considered that after 1975 there are more informative publications or reviews addressing the Glasgow-Pittsburgh Cerebral Performance Categories**

We have updated this reference in the manuscript.

**l. 424 - Ref [21] - please also provide the English translation**

We have provided the English translation for this reference.

**Table 1 - is "social precariousness" an alternative term for "homeless" as used in the manuscript? If so, use consistent wording in text and table.**

We used solely the term “social precariousness” in accordance with this comment.

**Table 3 - the high number of complications (21 in 37 patients) needs special attention in the discussion, also considering current international disputes on the added value of pre-hospital use of ECPR.**

We have made revisions to Table 2 concerning the count of complications. Furthermore, in Table 2, we have distinguished between complications that occurred in patients undergoing prehospital ECPR and those undergoing in-hospital ECPR.

**It may help the reader when mentioning the item "survival after 28 days" in table 3, that there were 7 resp 20 persons with pre-hospital resp in-hospital ECPR. The percentages in the column Cold air most likely need to be based on these numbers.**

We have made changes to Table 3 accordingly.
